# Notice of McPherson Hospital

**Published:** 1865-05

**Authors:** T. P. Robb

**Affiliations:** State Sanitary Commissioner


### NOTICE OF McPHERSON HOSPITAL.

**By Col. T. P. ROBB, State Sanitary Commissioner.**

McPherson Hospital occupies the building erected by the city for a city hospital, and used as such previous to the rebellion. It occupies a commanding site, and overlooks much of the ground made historical by the memorable "siege of Vicksburg." It is a commodious structure, but has been much improved since its occupation by us, especially in its interior arrangements. It is, and has been since its establishment, under the charge of Surgeon Powell, of the 72d Illinois Infantry Volunteers. Surgeon Powell has evidently, with a just and commendable pride in his profession and office, devoted all the powers of his cultivated mind to the making it "the Model Hospital," and to my mind he has fully succeeded. It surpasses anything of the kind that I have witnessed in all my experience. It seems to be perfect in all its parts and arrangements. The kitchen, bakery, store-room, and dining hall seem faultless, and would positively be viewed with envious eyes by our best and most fastidious housekeepers of the north, proverbial as that region is for its women of housekeeping propensities. The wards are neatly arranged with an eye single to the comfort of the inmates, and all the rooms are kept in a state of continual cleanliness, that would elicit the admiration of the most zealous devotee of the broom and scrubbing-brush. There is a place for everything and everything in its place. The most perfect order prevails. For the convalescents, (and in such a well ordered house they are numerous,) there is a reading-room, with its large and well selected library, bath-rooms, wash-room, and barber-shop. There is a billiard-room, with a good, though confiscated rebel, billiard table. Though not a judge of such articles, I presume it was "one of Phelan's best." In the yard are swings and other gymnastic apparatus, and a fine bowling-alley just being completed. These improvements have been made, for the most part, without cost to the government, by the aid of convalescent inmates, and show what can be accomplished by an energetic and intelligent surgeon, whose heart is in his work. No one, after spending an hour in the hospital, will doubt that all these things contribute much to the recovery of the patients. It is to be hoped that more of our surgeons, in charge of hospitals, will in like manner attempt the improvement of the surroundings and interior arrangements. In my opinion the trouble would be amply repaid in the increased comfort, contentedness, and speedy convalescence of the inmates.—*Report to State Sanitary Commission.*

